# Амиодарон-индуцированный тиреотоксикоз 2 типа: распространенность, сроки и предикторы развития

**DOI:** 10.14341/probl13348

**Published:** 2023-10-23

**Authors:** А. С. Ермолаева, В. В. Фадеев

**Affiliations:** Первый Московский государственный медицинский университет им. И.М. Сеченова (Сеченовский Университет); Первый Московский государственный медицинский университет им. И.М. Сеченова (Сеченовский Университет)

**Keywords:** амиодарон, щитовидная железа, амиодарон-индуцированный тиреотоксикоз 2 типа, предикторы

## Abstract

ОБОСНОВАНИЕ. Амиодарон занимает ведущую позицию в аритмологической практике в профилактике и купировании различных нарушений ритма сердца. Амиодарон — индуцированный тиреотоксикоз 2 типа — нередкое осложнение терапии препаратом, наиболее сложный вид дисфункции щитовидной железы как по тяжести клинических проявлений, так и с точки зрения понимания механизмов патогенеза, возможности дифференциальной диагностики и обеспечения эффективного лечения. В связи с увеличивающейся продолжительностью жизни населения, соответствующим ростом частоты нарушений ритма сердца проблема не теряет актуальности. Выявление предикторов, оценка и прогнозирование индивидуального риска развития данной патологии щитовидной железы — необходимость в повседневной клинической практике для принятия взвешенного решения при назначении антиаритмика, определении алгоритма дальнейшего динамического наблюдения пациента.ЦЕЛЬ. Оценить структуру амиодарон-индуцированной дисфункции щитовидной железы, распространенность, сроки и предикторы развития амиодарон-индуцированного тиреотоксикоза 2 типа в проспективном когортном исследовании.МАТЕРИАЛЫ И МЕТОДЫ. В исследовании приняли участие 124 пациента без нарушения функции щитовидной железы, впервые получившие терапию амиодароном. Оценка функционального состояния щитовидной железы производилась исходно, после назначения препарата первые 3 месяца 1 раз в месяц, в дальнейшем — каждые 3 месяца. Период наблюдения в среднем составил от 12 до 24 месяцев. Завершение наблюдения происходило при развитии амиодарон-индуцированной дисфункции щитовидной железы или отказе пациента от дальнейшего участия в исследовании. Для дифференциальной диагностики типа амиодарон-индуцированного тиреотоксикоза проводилось определение уровня антител к рецептору тиреотропного гормона, ультразвуковое исследование с доплерографией и сцинтиграфия щитовидной железы с пертехнетатом технеция. Оценивались вид и частота дисфункции щитовидной железы, время возникновения и предикторы амиодарон-индуцированного тиреотоксикоза 2 типа.РЕЗУЛЬТАТЫ. Структура амиодарон-индуцированной дисфункции щитовидной железы была представлена: в 19,3% (n=24) гипотиреозом, в 1,6% (n=2) тиреотоксикозом 1 типа, в 23,4% (n=29) тиреотоксикозом 2 типа. Медиана времени его развития составила 92,0 [69,0; 116,0] недель; средний срок бессобытийной выживаемости — 150,2±12,6 недель (95% ДИ: 125,5–175,0), медиана — 144±21,7 недель (95% ДИ: 101,4–186,6). Основными предикторами амиодарон-индуцированного тиреотоксикоза 2 типа являлись: возраст (ОШ=0,931, 95% ДИ: 0,895–0,968, р<0,001), ИМТ (ОШ=0,859, 95% ДИ: 0,762–0,967, р=0,012), время от начала терапии (ОШ=1,023, 95% ДИ: 1,008–1,038, р=0,003). Возраст ≤60 лет сопряжен с увеличением риска возникновения дисфункции в 2,4 раза (ОШ=2,352, 95% ДИ: 1,053–5,253, р=0,037), ИМТ≤26,6 кг/м2 — в 2,3 раза (ОШ=2,301, 95% ДИ: 1,025–5,165, р=0,043).ЗАКЛЮЧЕНИЕ. Полученные результаты позволяют персонализировано оценить риск развития амиодарон-индуцированного тиреотоксикоза 2 типа и определить адекватную тактику ведения пациента.

## Abstract (en)

**BACKGROUND:**

BACKGROUND: Amiodarone takes a leading position in arrhythmological practice in the prevention and relief of various cardiac arrhythmias. Type 2 amiodarone-induced thyrotoxicosis is a frequent side effect of the drug. It is the most complex type of thyroid dysfunction both in terms of the severity of clinical manifestations, and in terms of understanding the mechanisms of pathogenesis, possibility of differential diagnosis and providing effective treatment. Due to the increasing life expectancy of the population, corresponding increase in the frequency of cardiac arrhythmias, the problem does not lose its relevance. Identification of predictors, assessment and prediction of the individual risk of developing this thyroid pathology is a necessity in daily clinical practice for making a reasonable decision when prescribing the drug, determining the algorithm for further dynamic monitoring of the patient.

**AIM:**

AIM: To evaluate the structure of amiodarone-induced thyroid dysfunction, prevalence, time and predictors of development type 2 amiodarone-induced thyrotoxicosis in a prospective cohort study.

**MATERIALS AND METHODS:**

MATERIALS AND METHODS: The study involved 124 patients without thyroid dysfunction who received amiodarone therapy for the first time. Evaluation of the functional state of the thyroid gland was performed initially, after prescribing the drug for the first 3 months 1 time per month, in the future – every 3 months. The follow-up period averaged 12-24 months. The end of the observation occurred with the development of amiodaron-induced thyroid dysfunction or patient's refusal to further participate in the study. For the differential diagnosis of the type of amiodarone-induced thyrotoxicosis, the level of anti-TSH receptor antibodies and thyroid scintigraphy with technetium pertechnetate were determined. The type and frequency of thyroid dysfunction, time and predictors of development type 2 amiodarone-induced thyrotoxicosis were evaluated.

**RESULTS:**

RESULTS: The structure of amiodarone-induced thyroid dysfunction was represented by hypothyroidism in 19,3% (n=24), type 1 thyrotoxicosis in 1,6% (n=2), type 2 thyrotoxicosis in 23,4% (n=29). The median time of its development was 92,0 [69,0;116,0] weeks; the average period of common survival – 150,2±12,6 weeks (95% CI: 125,5–175,0), median – 144±21,7 weeks (95% CI: 101,4–186,6). The main predictors of type 2 amiodarone-induced thyrotoxicosis were: age (OR=0,931; 95% CI: 0,895–0,968; p<0.001), BMI (OR=0,859; 95% CI: 0,762–0,967; p=0,012), time from the start of amiodarone therapy (OR=1,023; 95% CI: 1,008–1,038; p=0,003). Age ≤60 years was associated with increased risk of the dysfunction by 2.4 times (OR=2,352; 95% CI: 1,053–5,253; p=0,037), BMI≤26,6 kg/m2 – 2,3 times (OR=2,301; 95% CI: 1,025–5,165; p=0,043).

**CONCLUSION:**

CONCLUSION: The results allow to personalized estimate the risk of type 2 amiodarone-induced thyrotoxicosis and determine the patient's management tactic.

## Обоснование

Нарушения ритма сердца — важная проблема здравоохранения, значительно увеличивающая риск сердечно-сосудистых осложнений и внезапной смерти, приводящая к снижению качества жизни, инвалидизации, высокой смертности, существенным экономическим затратам. Ведущая роль принадлежит фибрилляции предсердий, предполагаемая распространенность составляет 2–4%. В ближайшие десятилетия ожидается дальнейший ее рост, в основном в связи с увеличением продолжительности жизни населения и активизации поиска недиагностированных форм [1–3].

Амиодарон — высокоэффективный препарат для купирования жизнеугрожающих наджелудочковых и желудочковых аритмий, длительного поддержания синусового ритма. Считается фармакотерапией первой линии у пациентов со структурной патологией сердца, хронической сердечной недостаточностью, низкой фракцией выброса левого желудочка, имплантированными ресинхронизаторами, кардиоверторами-дефибрилляторами [2–5]. Частота его назначения среди других антиаритмических препаратов составляет: в России до 24% [6], США до 38% [7], Нидерландах до 41,8% [8], Китае до 53% [9], Великобритании до 61,8% [10].

Амиодарон — антиаритмический препарат III класса с высоким содержанием йода: 1 таблетка (200 мг) препарата содержит 74 мг йода, при метаболизме которого высвобождается около 7 мг йода в сутки, что во много раз превышает суточную потребность в элементе. Диспозиционная кинетика антиаритмика достаточно сложна. Ему свойственна вариабельная биодоступность (22–86%), биотрансформация с образованием фармакологически активного метаболита — дезэтиламиодарона, экстремально высокая липофильность, широкое распределение во многие органы и ткани (66 л/кг массы тела), высокая аффинность к ним. Амиодарон и дезэтиламиодарон обладают способностью активно накапливаться некоторыми тканями организма (жировой тканью, печенью, легкими, в меньшей степени скелетной мускулатурой, почками, сердцем, мозгом). Характерна очень медленная элиминация, в основном с желчью с некоторой степенью кишечно-печеночной рециркуляции и незначительной почечной экскрецией, период полувыведения составляет от 22 до 100 дней. Таким образом, сам антиаритмик и йодированные продукты его метаболизма могут сохраняться в организме долгое время после отмены препарата [11–15].

Влияние амиодарона на функцию щитовидной железы происходит в двух направлениях: нарушение синтеза и метаболизма тиреоидных гормонов (блок органификации йода (эффект Вольффа-Чайкоффа) [16], ингибирование 5-дейодинирования тироксина и реверсивного трийодтиронина [17], взаимодействие с ядерными рецепторами [18]) и прямое цитотоксическое действие на тиреоциты [19]. Первые 3 месяца терапии часто наблюдается обычно преходящее повышение уровня тиреотропного гормона (ТТГ) в сыворотке крови. Верхнереференсный/повышенный уровень общего и свободного тироксина (свТ4), реверсивный Т3, низкореференсный/пониженный уровень общего и свободного трийодтиронина (свТ3) может сохраняться длительно, даже в течение нескольких месяцев после отмены препарата, что обусловлено подавлением активности 5-дейодиназы I типа [20].

Наиболее тяжелым видом дисфункции щитовидной железы является амиодарон-индуцированный тиреотоксикоз (АмИТ): АмИТ1 — результат феномена Йод-Базедова, как правило возникает у пациентов с латентной патологией щитовидной железы (болезнь Грейвса, функциональная автономия), характеризуется гиперпродукцией тиреоидных гормонов; АмИТ2 — деструктивный тиреоидит, развивающийся в основном при интактной щитовидной железе, при котором происходит высвобождение ранее синтезированных гормонов в кровоток [21][22]. Дифференциация на патогенетические типы важна для адекватного терапевтического подхода. Для АмИТ2, как правило, характерен низкий уровень антител к рецептору тиреотропного гормона (АТ-рТТГ), отсутствие гиперваскуляризации по данным цветового доплеровского картирования, низкий захват пертехнетата (99mTcO4) или технетрила (99mTc-sestaMIBI) по результатам сцинтиграфии. В последние годы предпочтение отдается технетрилу поскольку отсутствует искажение захвата при дефекте йодидной органификации (диффузия осуществляется через клеточную мембрану, минуя натрий-йодидный симпортер), а накопление радиофармпрепарата происходит при высоком митохондриальном потенциале клеток [11][23][24].

В большинстве ретроспективных исследований представлена суммарная распространенность АмИТ с указанием преобладания тиреотоксикоза 2 типа, варьирующая в широких пределах от 4,0–13,6% [25–28] до 20,5–43,9% с учетом субклинических форм [29–31]. Возможно, она обусловлена различиями в диагностических критериях АмИТ, частоте мониторинга функции щитовидной железы, географических различиях потребления йода, включением пациентов с умеренными функциональными нарушениями щитовидной железы [26][33]. Увеличение распространенности АмИТ2 за последние десятилетия обусловлено большим вниманием к данной проблеме, тщательным обследованием и отбором кандидатов на лечение, избеганием назначения препарата пациентам с латентными нарушениями функции щитовидной железы, а также ориентированием пациентов на регулярный мониторинг [34]. Распространенность манифестного АмИТ в России составляет 5,8–15,8%, по АмИТ2 статистики практически не существует [35–38].

В настоящее время специфические предикторы АмИТ2 не известны [27]. Данные о влиянии дозы амиодарона и длительности терапии противоречивы [29][39]. Наличие дилатационной кардиомиопатии, саркоидоза сердца, тяжелого врожденного порока, хронической сердечной недостаточности III–IV функционального класса (NYHA) сопряжено с большей частотой амиодарон-индуцированной тиреопатии, однако у данной категории пациентов высока вероятность получения и большей накопительной дозы препарата. Возможно, долгосрочный риск дисфункции щитовидной железы зависит от кумулятивной дозы антиаритмика в течение первого года лечения [28][33][40][41]. Прогностическими факторами развития АмИТ2 могут служить возраст [24][27], низкий индекс массы тела [28], а также наличие хронической обструктивной болезни легких [42]. Несоблюдение алгоритма наблюдения при приеме амиодарона — определение функционального состояния щитовидной железы до назначения препарата, каждые 6 месяцев во время лечения и в течение 12–24 месяцев после его отмены затрудняет своевременную диагностику и лечение дисфункции щитовидной железы [2][22][43].

## ЦЕЛЬ ИССЛЕДОВАНИЯ

Оценить структуру амиодарон-индуцированной дисфункции щитовидной железы, распространенность, сроки и предикторы развития АмИТ2 в проспективном когортном исследовании у пациентов, впервые получивших антиаритмическую терапию амиодароном.

## МАТЕРИАЛЫ И МЕТОДЫ

## Место и время проведения исследования

Место проведения. Исследование проведено на базе клиник ФГАОУ ВО Первый МГМУ им. И.М. Сеченова Минздрава России (Сеченовский Университет).

Время исследования. Наблюдение пациентов осуществлялось с января 2009 по август 2013 гг.

## Изучаемые популяции (одна или несколько)

Использовался сплошной способ формирования выборки. Изучалась одна популяция: пациенты, проживающие в Москве и Московской области, без нарушения функции щитовидной железы, с наличием или отсутствием структурных изменений, впервые получившие терапию амиодароном.

Критериями включения являлись: возраст 18–85 лет, эутиреоз до назначения амиодарона, минимальный период наблюдения на фоне приема амиодарона — 12 месяцев, при замене антиаритмика раньше этого времени (минимальный срок приема амиодарона 1 месяц) — дальнейший мониторинг функционального состояния щитовидной железы в течение 12 месяцев, отсутствие в анамнезе нарушений функции щитовидной железы, подписанное информированное согласие на участие в исследовании.

Критерии невключения: терапия амиодароном в анамнезе, ангиография с использованием йодсодержащего контрастного вещества в течение последних 6 месяцев, прием препаратов лития, интерферона, интерлейкина-2, моноклональных антител, ингибиторов протеинкиназ, выраженная почечная и печеночная недостаточность, психические расстройства, беременность, период лактации.

## Дизайн исследования

Проведено проспективное исследование, включавшее 124 пациента (69 мужчин и 55 женщин) без нарушения функции щитовидной железы, которым впервые был назначен амиодарон. Исходно и после назначения первые 3 месяца 1 раз в месяц и далее каждые 3 месяца проводилось исследование уровней: ТТГ, свТ4 и свТ3, антител к тиреоидной пероксидазе (АТ-ТПО); выполнялось УЗИ щитовидной железы с доплерогафией — исходно и далее с интервалом 12 месяцев. Период наблюдения в среднем составил от 12 до 24 месяцев. Пациентам с АмИТ проводилось исследование уровня АТ-рТТГ и сцинтиграфия щитовидной железы с пертехнетатом технеция. Пациенты с АмИТ2 стали участниками проспективного исследования по оценке эффективности различных вариантов терапии глюкокортикоидами.

## Описание медицинского вмешательства (для интервенционных исследований)

Клинические методы обследования включали сбор анамнеза (сердечно-сосудистая и сопутствующая патологии, отсутствие/наличие курения), антропометрических данных, определение функционального состояния щитовидной железы до назначения амиодарона: исследование уровней ТТГ, свТ4, свТ3, АТ-ТПО; проведение УЗИ щитовидной железы с доплерографией; в дальнейшем — на фоне приема препарата согласно дизайну исследования; регистрация суточной дозы амиодарона и длительности приема. Для морфологической верификации узловых образований проводилась тонкоигольная аспирационная биопсия. При развитии АмИТ — определение уровня АТ-рТТГ и сцинтиграфия щитовидной железы с технецием-99м-пертехнетатом. Йодный статус пациентов был определен по данным Глобальной сети по йоду (Iodine Global Network) и литературных источников [44–46], медианная концентрация йода в моче не определялась.

## Методы

Уровни ТТГ (референс 0,4–4,0 мкМЕ/мл), свТ4 (референс 11,5–23,2 пмоль/л) определялись иммунохемилюминесцентным методом с помощью набора Immulite (США), свТ3 (референс 3,5–6,5 пмоль/л) — иммунохемилюминесцентным методом с помощью набора Вayer-ACS:180 (Германия). АТ-ТПО — иммуноферментным методом с помощью набора «Хема-Медика» (Россия), референс 0–60 МЕ/мл; АТ-рТТГ — радиорецепторным методом с помощью набора “CIS Bio International” (Франция), референс 0–1 МЕ/л. УЗИ щитовидной железы проводили аппаратом Voluson 730 Pro (General Electric, США). Увеличенным считался объем >18 мл у женщин и >25 мл у мужчин. Тонкоигольная аспирационная биопсия узловых образований выполнялась иглой для внутримышечных инъекций 21G под контролем УЗИ c последующим цитологическим исследованием в патоморфологическом отделении: окрашиванием мазков по методу Май-Грюнвальда-Гимзы и микроскопическим исследованием на аппарате Leica DM 2500 (Leica Microsystems GmbH, Германия). Сцинтиграфия щитовидной железы с пертехнетатом технеция-99mTcO4 производилась с помощью вращающейся гамма-камеры GE 400T (General Electric, Бостон, Массачусетс, США).

Эутиреоидная гипертироксинемия регистрировалась при повышенном уровне свТ4 в сочетании с низкореференсным/пониженным значением свТ3 и референсным уровнем ТТГ. Транзиторный гипотиреоз устанавливался при временном (не более 6 месяцев) повышении уровня ТТГ>4,0 мкМЕ/мл с последующим возвращением в референсный диапазон; субклинический — при значениях ТТГ>4,0<10,0 мкМЕ/мл и манифестный — при значениях ТТГ>10,0 мкМЕ/мл, сохраняющихся более 6 месяцев. Манифестный АмИТ регистрировался при снижении уровня ТТГ<0,4 мкМЕ/мл, превышении значений свТ4>23,2 пмоль/л, референсном или свТ3>6,5 пмоль/л в сыворотке крови; субклинический АмИТ — снижении уровня ТТГ<0,4 мкМЕ/мл и референсных значениях свТ4 и свТ3. АмИТ1 подтверждался наличием гиперваскуляризации при доплерографии, повышенным захватом пертехнетата технеция по данным сцинтиграфии (>1,8%); АмИТ2 — отсутствием гиперваскуляризации, АТ-рТТГ (<1 МЕ/л) и низким захватом пертехнетата технеция (<1%).

Наблюдение завершалось при развитии амиодарон-индуцированной дисфункции щитовидной железы или отказе пациента от дальнейшего участия в исследовании. Оценивались вид и частота дисфункции, время возникновения и предикторы АмИТ2.

## Статистический анализ

Проверка количественных данных на нормальность распределения произведена с помощью критерия Колмогорова-Смирнова и по величине асимметрии и эксцесса. Описательные статистики для количественных переменных представлены в виде медианы, интерквартильного диапазона (Mе [Q1; Q3]), для категориальных переменных — в процентах. Статистическая значимость различий между группами для количественных данных определялась с помощью критерия Краскела-Уоллиса и последующим роst-hос анализом с применением поправки Бонферрони; апостериорные парные сравнения — с помощью критерия Манна-Уитни. Изменения количественных показателей в связанных выборках оценивались с помощью критерия Уилкоксона и Фридмана с последующим post-hoc анализом с поправкой на множественность сравнений. Анализ номинальных данных производился с использованием χ² Пирсона и точного критерия Фишера. Для оценки влияния факторов на выживаемость применялся метод Каплана-Мейера с использованием лог-ранк критерия Мантеля-Кокса. Построение прогностических моделей производилось методом бинарной логистической регрессии с последующим ROC-анализом и регрессии Кокса. Различия считались статистически значимыми при значении p<0,05. Статистический анализ данных осуществлен при помощи пакета статистических программ SPSS v. 26 (SPSS, Chicago, IL, USA).

## Этическая экспертиза

Протокол исследования рассмотрен и одобрен на заседании комитета по этике при ассоциации медфармвузов при ПМГМУ им. И.М. Сеченова (протокол №10-08 от 11 декабря 2008 г.).

## Результаты

В исследовании приняли участие 124 пациента: 69 (55,6%) мужчин и 55 (44,4%) женщин в возрасте от 23 до 85 лет, госпитализированных по поводу нарушений ритма сердца. В 46% (n=57) случаев регистрировалась фибрилляция предсердий (рис. 1), у трети пациентов (43% (n=53)) отмечалась декомпенсация хронической сердечной недостаточности, у 21% (n=26) — сниженная фракция выброса левого желудочка, у 19,4% (n=24) — легочная гипертензия.

**Figure:**
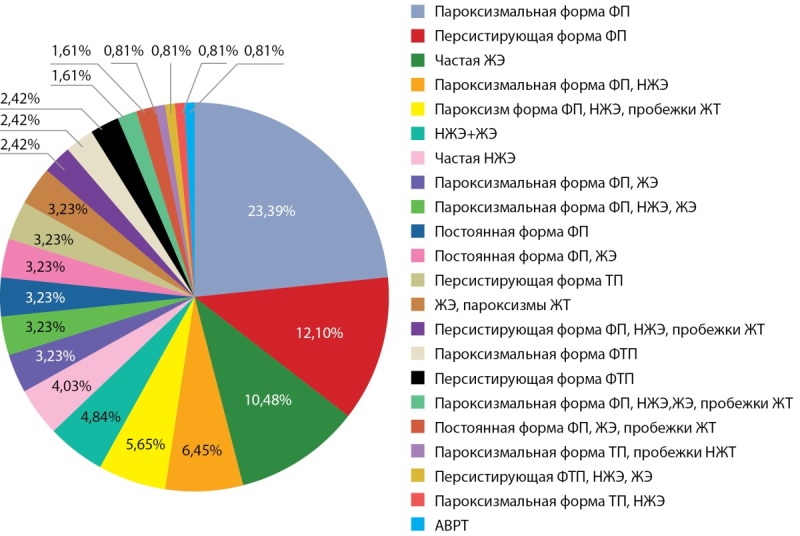
Рисунок 1. Виды нарушений ритма сердца при назначении амиодарона. Примечание: НЖЭ — наджелудочковая экстрасистолия; ФП — фибрилляция предсердий; ТП — трепетание предсердий; ФТП — фибрилляция и трепетание предсердий, ЖЭ — желудочковая экстрасистолия; ЖТ — желудочковая тахикардия; АВРТ — атриовентрикулярная реципрокная тахикардия.

Гендерных различий по возрасту, индексу массы тела (ИМТ), скорости клубочковой фильтрации, суточной и кумулятивной дозе амиодарона, длительности приема, имеющейся сердечно-сосудистой патологии выявлено не было. Большая частота узлового/многоузлового коллоидного зоба встречалась у женщин (р=0,002), однако различия по частоте амиодарон-индуцированной дисфункции по сравнению с мужчинами отсутствовали.

По приему амиодарона и соответствующему периоду наблюдения пациенты были распределены на 5 групп: группа 1 — длительность приема менее 12 месяцев с заменой на другой антиаритмический препарат и дальнейшим наблюдением в течение года; группа 2 — прием и наблюдение в течение 12 месяцев; группа 3 — прием и наблюдение в течение 18 месяцев; группа 4 — прием и наблюдение в течение 24 месяцев; группа 5 — прием и наблюдение более 24 месяцев. Характеристика пациентов по группам представлена в табл. 1.

**Table:** Таблица 1. Характеристика пациентов Примечание: ХНЗЛ — хронические неспецифические заболевания легких; СКФ — скорость клубочковой фильтрации; ХСН — хроническая сердечная недостаточность; ФВЛЖ — фракция выброса левого желудочка; СДЛА — систолическое давление в легочной артерии; ЩЖ — щитовидная железа; Ам — амиодарон; свТ4максАмИТ2 — максимальный уровень свТ4 при АмИТ2; свТ3максАмИТ2 — максимальный уровень свТ3 при АмИТ2; свТ4макс/свТ3макс — соотношение максимальных значений свободных фракций Т4 и Т3 при АмИТ2; ИЗ99mTcO4 — индекс захвата пертехнетата технеция; АмИГ — амиодарон-индуцированный гипотиреоз. *различия показателей между группами статистически значимы (р<0,05).

Параметр	Все	Замена Ам (1)	12 мес (2)	18 мес (3)	24 мес (4)	более 24 мес (5)	р1-5-value
Число пациентов, n (%):	124	11 (8,9)	47 (37,9)	16 (12,9)	37 (29,8)	13 (10,5)	
Пол:							0,794
мужской	69 (55,6)	6 (54,5)	29 (61,7)	7 (43,7)	20 (54,1)	7 (53,8)
женский	55 (44,4)	5 (45,5)	18 (38,3)	9 (56,3)	17 (45,9)	6 (46,2)
Возраст	62,0[ 55,5; 71,0]	54,0[ 44,5; 67,5]	61,0[ 57,0; 70,0]	63,5[ 58,0; 72,5]	69,8[ 60,6; 80,9]	60,0[ 56,0; 67,0]	0,389
Курение	33 (26,6)	6 (54,5)	15 (31,9)	3 (18,8)	8 (21,6)	1 (7,7)	0,094
Заболевание легких (ХНЗЛ)	18 (14,5)		9 (20,0)	3 (18,8)	4 (10,8)	2 (15,4)	0,536
ИМТ, кг/м²	28,4[ 25,2; 31,4]	26,5[ 25,5; 29,3]	30,1[ 26,3; 34,1]	28,3[ 23,4; 32,0]	28,5[ 25,7; 30,7]	26,6[ 24,2; 29,7]	0,209
СД 2 типа	25 (20,2)	2 (18,2)	11 (23,4)	4 (25)	5 (13,5)	3 (23,1)	0,800
Повышение трансаминаз	23 (18,5)	1 (9,1)	8 (17,0)	4 (25)	8 (21,6)	2 (15,4)	0,830
СКФCKD-EPI, мл/мин/1,73 м²	69,6[ 59,6; 82,1]	76,6[ 68,3; 97,3]	65,8[ 58,0; 81,9]	73,7[ 58,5; 88,1]	68,8[ 58,7; 80,5]	71,8[ 62,7; 77,2]	0,555
Нарушения ритма:							0,039*
предсердные	75 (60,5)	6 (54,5)	24 (51,1)	9 (56,3)	29 (78,4)	7 (53,8)
желудочковые	16 (12,9)	4 (36,4)	5 (10,6)	2 (12,5)	4 (10,8)	1 (7,7)
комбинированные	33 (26,6)	1 (9,1)	18 (38,3)	5 (31,3)	4 (10,8)	5 (38,5)
ХСН, NYHA:							0,227
II ФК	47 (37,9)	5 (45,5)	18 (38,3)	7 (43,8)	12 (32,4)	5 (38,5)
III ФК	6 (4,8)	2 (18,2)	3 (6,4)	1 (6,3)		
ФВЛЖ, %	57,0[ 51,0; 61,0]	53,0[ 33,0; 59,5]	58,0[ 50,0; 61,0]	54,5[ 50,0; 56,0]	59,0[ 54,0; 63,0]	59,0[ 54,0; 62,0]	0,055
ХСНФВЛЖ:							0,388
c промежуточной	14 (11,3)		7 (14,9)	2 (12,5)	3 (8,1)	2 (15,4)
c низкой	12 (9,7)	4 (36,4)	5 (10,6)	1 (6,3)	2 (5,4)	
СДЛА, мм рт.ст.	26,0[ 24,0; 30,0]	28,0[ 25,0; 34,5]	28,0[ 24,0; 30,0]	26,5[ 24,5; 35,5]	25,0[ 22,0; 28,0]	25,0[ 23,0; 27,0]	0,187
Порок сердца	21 (16,9)	2 (18,2)	7 (14,9)	1 (6,3)	7 (18,9)	4 (30,8)	0,515
Миокардит	14 (11,3)	3 (27,3)	4 (8,5)	2 (12,5)	1 (2,7)	4 (30,8)	0,028*
Кардиомиопатия	10 (8,1)	1 (9,1)	5 (10,6)		3 (8,1)	1 (7,7)	0,765
ТТГисходно, мкМЕ/мл	2,2[ 1,8; 3,0]	2,1[ 1,7; 2,3]	2,3[ 1,6; 3,5]	1,9[ 1,8; 3,0]	2,3[ 1,9; 2,6]	2,8[ 1,9; 3,0]	0,728
свТ4исходно, пмоль/л	16,6[ 14,9; 18,7]	16,4[ 15,0; 17,5]	16,6[ 15,1; 18,6]	16,6[ 14,4; 18,8]	16,4[ 14,5; 18,6]	17,8[ 15,1; 19,6]	0,795
свТ3исходно, пмоль/л	4,7[ 4,3; 5,2]	4,8[ 4,4; 5,1]	4,7[ 4,3; 5,2]	4,4[ 4,3; 5,2]	4,8[ 4,4; 5,1]	4,8[ 4,5; 5,0]	0,816
АТ-ТПО, МЕ/мл	14,0[ 11,0; 20,5]	11,0[ 9,0; 13,5]	16,0[ 13,0; 21,5]	12,0[ 10,0; 20,0]	14,0[ 11,0; 23,5]	14,0[ 11,0; 17,0]	0,057
Исходная структурная патология ЩЖ:							0,903
нет	94 (75,8)	9 (81,8)	36 (76,6)		25 (67,6)	1 (7,7)
узловой зоб	10 (8,1)		5 (10,6)	14 (87,5)	3 (8,1)	1 (7,7)
многоузловой зоб	15 (12,1)	2 (18,2)	4 (8,5)	1 (6,3)	7 (18,9)	1 (7,7)
диффузный зоб	5 (4,0)		2 (4,3)	1 (6,3)	2 (5,4)	1 (7,7)
Объем ЩЖ, мл	15,4[ 11,0; 19,2]	15,0[ 10,1; 17,2]	15,8[ 13,0; 19,9]	14,9[ 12,0; 21,0]	15,9[ 11,3; 18,8]	12,0[ 9,9; 15,7]	0,317
Длительность приема Ам, недели	73,5[ 52,0; 104,0]	21,0[ 13,0; 26,0]	52,0[ 52,0; 52,0]	78,0[ 73,5; 78,0]	104,0[ 104,0; 104,0]	153,0[ 116,0; 156,0]	<0,001*
Суточная доза Ам, мг	200[ 200; 300]	200[ 200; 300]	200[ 200; 300]	250[ 200; 350]	200[ 200; 200]	200[ 200; 200]	0,099
Кумулятивная доза Ам, г	135,8[ 73,0; 145,6]	36,4[ 23,8; 36,4]	73,0[ 72,8; 109,4]	118,7[ 109,2; 163,8]	145,6[ 145,6; 145,6]	203,8[ 162,4; 218,4]	<0,001*
Время развития АмИТ2 от начала приема Ам, недели	92,0[ 69,0; 116,0]	86,0[ 79,5; 89,0]	43,0[ 33,0; 48,0]	69,0[ 65,0; 78,0]	98,0[ 92,5; 101,0]	156,0[ 128,0; 205,0]	<0,001*
свТ4максАмИТ2, пмоль/л	30,9[ 25,6; 40,5]	27,8[ 25,5; 31,2]	34,2[ 26,7; 38,3]	27,6[ 23,0; 56,9]	41,8[ 31,3; 51,1]	31,2[ 26,3; 42,3]	0,789
свТ3максАмИТ2, пмоль/л	7,2[ 6,5; 8,9]	6,6[ 6,6; 7,1]	7,1[ 6,1; 8,1]	6,9[ 6,4; 12,4]	9,1[ 7,3; 10,7]	7,9[ 6,7; 10,3]	0,617
свТ4макс/свТ3макс	4,0[ 3,6; 4,4]	4,2[ 3,9; 4,4]	4,8[ 4,3; 5,1]	3,8[ 3,6; 4,5]	4,1[ 3,8; 4,8]	3,9[ 3,8; 4,1]	0,260
АТ-рТТГАмИТ2, МЕ/л	0,6[ 0,4; 0,6]	0,4[ 0,4; 0,5]	0,4[ 0,3; 0,4]	0,6[ 0,5; 0,6]	0,4[ 0,4; 0,7]	0,6[ 0,4; 0,6]	0,411
Объем ЩЖАмИТ2, мл	17,0[ 12,9; 22,9]	9,7[ 9,4; 13,9]	21,8[ 15,5; 29,9]	15,5[ 12,7; 22,8]	19,3[ 17,9; 23,5]	13,8[ 13,0; 21,3]	0,249
ИЗ99mTcO4, %	0,4[ 0,4; 0,6]	0,6[ 0,5; 0,7]	0,5[ 0,3; 0,6]	0,6[ 0,4; 0,6]	0,2[ 0,2; 0,4]	0,4[ 0,1; 0,6]	0,208
Эутиреоз	75 (60,5)	7 (63,6)	32 (68,1)	8 (50,0)	24 (64,9)	4 (30,8)	0,142
Эутиреоидная гипертироксинемия	8 (6,5)		3 (6,4)		3 (8,1)	2 (15,4)	0,490
Транзиторный гипотиреоз	27 (21,8)	4 (36,4)	11 (23,4)	3 (18,8)	8 (21,6)	2 (15,4)	0,385
АмИГсубклинический	18 (14,5)	1 (9,1)	4 (8,5)	3 (18,8)	8 (21,6)	2 (15,4)	0,399
АмИГманифестный	6 (4,8)		5 (10,6)		1 (2,7)		0,425
АмИТ1	2 (1,6)	1 (9,1)	1 (2,1)				0,287
АмИT2субклинический	7 (5,6)	1 (9,1)	1 (2,1)	2 (12,5)	1 (2,7)	2 (15,4)	0,930
АмИТ2манифестный	22 (17,7)	2 (18,2)	3 (6,4)	4 (25,0)	6 (16,2)	7 (53,8)	0,938
АмИТ2	29 (23,4)	3 (27,3)	4 (8,5)	6 (37,5)	7 (18,9)	9 (69,2)	<0,001*

Различия между группами отмечались по виду и частоте нарушений сердечного ритма (p=0,041): преобладание комбинированных (предсердные и желудочковые) нарушений в группе 2 по сравнению с группой 4 (р=0,044); частоте миокардита (р=0,028): меньшее число случаев в группе 4 по сравнению с группами 1 и 5 (р1-4=0,048, р4-5=0,037); длительности приема амиодарона (р<0,001): меньшая в группах 1 и 2 по сравнению с группами 3–5 (р1-3,4,5<0,001, р2-3=0,017, р2-4,5<0,001), в группе 3 по сравнению с группой 5 (р=0,001); кумулятивной дозе (р<0,001): меньшая в группах 1 и 2 по сравнению с группами 3–5 (р1-3,4,5<0,001, р2-3=0,035, р2-4,5<0,001), в группе 3 по сравнению с группой 5 (р=0,048); времени развития АмИТ2 от начала приема антиаритмика (р<0,001): меньшее в группах 2 и 3 по сравнению с группой 5 (р2-5<0,001, р3-5=0,001); а также по частоте развития АмИТ2 (p<0,001): меньшая в группе 2 по сравнению с группами 3 и 5 (р2-3=0,020, р2-5<0,001), в группе 4 по сравнению с группой 5 (р=0,004).

Наименьшая длительность приема амиодарона была в группе 1, медиана составила 21,0 [ 13,0; 26,0] неделю, время возникновения АмИТ2 от отмены препарата — 47,0 [ 42,5; 49,5] недель. Зависимость риска развития АмИТ2 от отмены амиодарона оценена с помощью лог-ранк критерия Мантеля-Кокса была статистически незначимой (р=0,062): медиана срока возникновения АмИТ2 в 1 группе составила 86,0±9,5 недели (95% ДИ: 67,4–104,6), среднее время — 86,3±3,7 недели (95% ДИ: 79,1–93,6), у продолжавших прием антиаритмика медиана составила 144±21,8 недели (95% ДИ: 101,2–186,7), среднее время — 152,9±13,1 недели (95% ДИ: 127,3–178,5) (рис. 2). Медиана срока общей выживаемости составила 144±21,7 недели (95% ДИ: 101,4–186,6), среднее время — 150,2±12,6 недели (95% ДИ: 125,5–175,0) (рис. 3).

**Figure:**
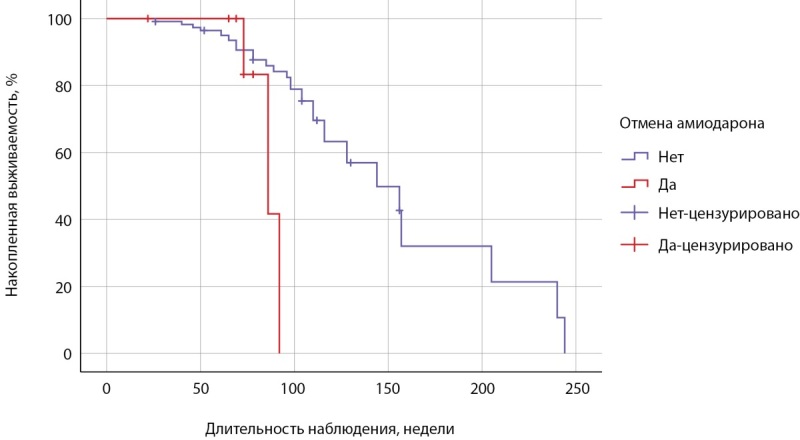
Рисунок 2. Кривая Каплана-Мейера, характеризующая бессобытийную выживаемость при отмене амиодарона.

**Figure:**
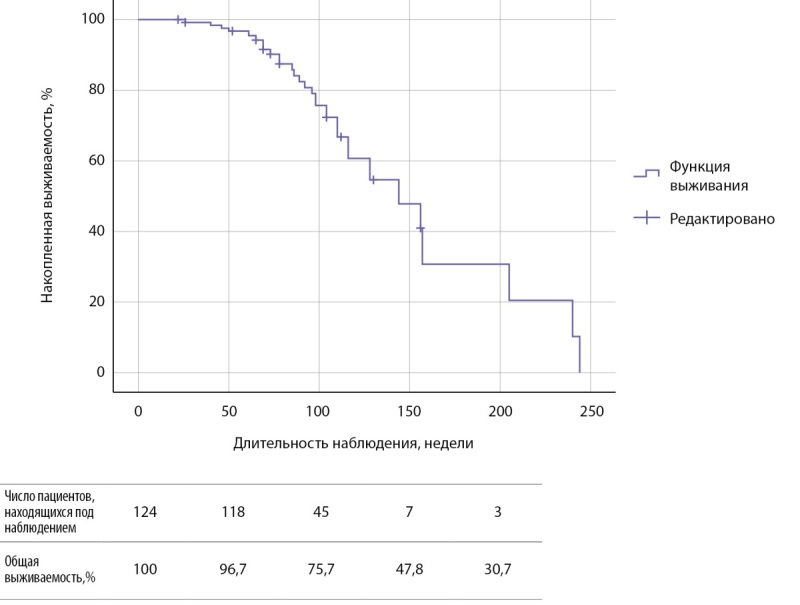
Рисунок 3. Кривая Каплана-Мейера, характеризующая общую выживаемость пациентов.

Динамика уровня ТТГ, свободных фракций тиреоидных гормонов и АТ-ТПО на фоне приема амиодарона представлена в табл. 2. Исходный низкореференсный уровень ТТГ (<1 мкМЕ/мл) отмечался в 4,0% (5/124) случаев, не влиял на развитие АмИТ2 (р=0,333). 87,1% (108/124) пациентов принимали амиодарон в течение года, 36,3% (46/124) — в течение двух лет. Статистически значимое увеличение уровня ТТГ, свТ4 и снижение свТ3 по сравнению с исходным значением в этих периодах наблюдения регистрировалось во всех временных точках мониторинга (р<0,001). Изменение объема щитовидной железы на фоне приема амиодарона не отмечалось (р0-12мес=0,378; р0-12-24мес=0,218).

**Table:** Таблица 2. Динамика уровня ТТГ, свободных фракций тиреоидных гормонов, АТ-ТПО на фоне приема амиодарона

Параметр	Исходно n=124	1 мес n=124	2 мес n=123	3 мес n=123	6 мес n=117	9 мес n=111	12 мес n=108	15 мес n=66	18 мес n=62	21 мес n=49	24 мес n=46
ТТГ, мкМЕ/мл	2,2[ 1,8; 3,0] 0,5–3,9	3,1[ 2,2; 4,0] 0,4–20,0	3,2[ 2,3; 4,1] 0,5–24,1	3,0[ 2,4; 4,3] 0,6–39,3	3,0[ 2,2; 4,0] 0,03–43,3	3,1[ 2,4; 3,8] 0,05–10,1	3,1[ 2,3; 3,9] 0,01–9,5	3,0[ 2,2; 4,0] 0,01–9,3	3,1[ 2,0; 3,9] 0,01–8,7	3,1[ 2,2; 3,6] 0,01–7,6	3,1[ 2,0; 3,6] 0,02–8,1
свТ4, пмоль/л	16,5[ 14,9; 18,6] 11,4–22,9	18,5[ 16,4; 20,2] 9,7–27,2	18,6[ 17,0; 19,7] 9,4–25,3	18,8[ 17,6; 20,0] 5,0–27,0	19,0[ 17,2; 20,2] 5,8–37,3	18,8[ 17,8; 19,7] 10,7–29,0	18,8[ 17,6; 19,8] 8,32–38,6	19,2[ 18,2; 19,8] 12,6–62,8	19,2[ 17,8; 19,9] 12,3–34,63	18,6[ 18,1; 19,6] 13,2–100,0	18,6[ 17,9; 19,6] 13,0–21,0
свТ3, пмоль/л	5,1[ 4,6; 5,4] 3,6–6,0	4,7[ 4,2; 4,9] 3,5–5,4	4,4[ 4,1; 4,7] 3,4-5,6	4,4[ 4,1; 4,8] 3,4–5,6	4,4[ 4,1; 4,7] 3,3–7,9	4,4[ 4,2; 4,8] 3,4–6,7	4,3[ 4,1; 4,8] 3,5–8,3	4,5[ 4,2; 4,9] 3,5–12,4	4,5[ 4,2; 4,9] 3,7–7,6	4,4[ 4,2; 4,8] 3,4–27,8	4,5[ 4,3; 4,7] 3,5–5,4
АТ-ТПО, МЕ/мл	14,0[ 11,0; 20,5] 4,0–662,0	15,0[ 12,0; 24,0] 6,0–820,0	16,0[ 13,0; 21,5] 4,0–1077,0	15,0[ 13,0; 22,0] 6,0–580,0	16,0[ 13,0; 23,0] 6,0–616,0	16,5[ 14,0; 20,5] 8,0–1000,0	16,0[ 14,0; 21,0] 8,0–814,0	16,5[ 13,0; 24,0] 5,0–736,0	17,0[ 13,5; 24,0] 10,0–627,0	16,5[ 13,5; 23,5] 11,0–753,0	18,0[ 14,0; 23,0] 10,0–658,0

Частота развития АмИТ2 в течение первого года наблюдения составила 3,2% (4/124), второго — 12,9% (16/24), третьего — 4,0% (5/124). Исходная структурная патология щитовидной железы регистрировалась в 27,6% (8/29) случаев: в 13,8% (4/29) — узловой коллоидный зоб, в 3,4% (1/29 — многоузловой коллоидный зоб, в 10,3% (3/29) — диффузный зоб. При цветовом доплеровском картировании отсутствовала гиперваскуляризация паренхимы, интранодулярный кровоток; у трех пациентов отмечался перинодулярный кровоток. Длительность приема амиодарона в группе АмИТ2 варьировала от 26 до 244 недель (89,0 [ 61,0; 110]). Кумулятивная доза статистически значимо была меньшей у пациентов с ХСН II–III ФК (11/29) по сравнению с пациентами без ХСН (р=0,021), диапазон вариабельности составил 36,4–512,4 г (137,2 [ 109,2; 168,0]). Медиана максимальных значений свТ4 составила 31,2 [ 25,3; 42,3], минимум 19,7, максимум 124,7 пмоль/л; свТ3 — 7,3 [ 6,5; 10,1], минимум 5,3, максимум 30,8 пмоль/л. Тяжесть тиреотоксикоза условно оценивалась по максимальным значениям свободных фракций тиреоидных гормонов. У 69,0% (20/29) пациентов отмечалось легкое течение АмИТ2 (свТ4<40; свТ3<10 пмоль/л), 24,1% (7/29) — умеренное (свТ4 40–80; свТ3 10–15 пмоль/л), 6,9% (2/29) — тяжелое (свТ4>80; свТ3>15 пмоль/л).

При оценке факторов риска развития АмИТ2 (параметры, указанные в табл. 1) статистически значимыми являлись: возраст начала терапии амиодароном (ОШ=0,945, 95% ДИ: 0,913–0,979; R²=0,129; р=0,001), ИМТ (ОШ=0,869, 95% ДИ: 0,785–0,963; R²=0,098 р=0,004), длительность приема препарата (ОШ=1,015, 95% ДИ: 1,003–1,026; R²=0,102; р=0,003), время от начала терапии (ОШ=1,020, 95% ДИ: 1,007–1,033; R²=0,138; р<0,001), наличие миокардита (ОШ=8,1, 95% ДИ: 2,450–26,780; R²=0,143; р<0,001).

Согласно полученной многофакторной модели бинарной логистической регрессии (R²=0,358, р<0,001) увеличение возраста на 1 год уменьшает шансы АмИТ2 в 1,07 раза, увеличение ИМТ на 1 кг/м² уменьшает шансы АмИТ2 в 1,16 раза, увеличение времени от начала терапии антиаритмиком увеличивает шансы АмИТ2 в 1,02 раза. Статистическая значимость длительности приема препарата (р=0,994) и миокардита (р=0,428) как предикторов АмИТ2 не подтвердилась. Характеристика предикторов представлена в табл. 3. Диагностическая значимость прогностической модели оценена с помощью метода ROC-кривых: площадь под ROC-кривой составила 0,818±0,049 с 95% ДИ: 0,722–0,913, пороговое значение функции в точке cut-off составило 0,195. Значения функции, равные или превышающие данное значение, соответствовали прогнозу развития АмИТ2, чувствительность — 79,3%, специфичность — 70,5% (рис. 4).

**Table:** Таблица 3. Предикторы АмИТ2, результаты бинарной логистической регрессии (метод исключения по Вальду) * влияние предиктора статистически значимо (р <0,05).

Параметр	ОШ	95% ДИ	р
Возраст	0,931	0,895–0,968	<0,001*
ИМТ	0,859	0,762–0,967	0,012*
Время от начала терапии амиодароном	1,023	1,008–1,038	0,003*

**Figure:**
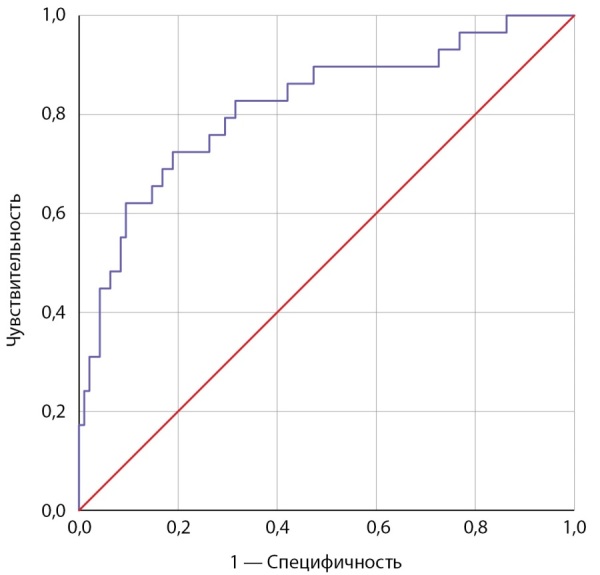
Рисунок 4. ROC-кривая вероятности развития АмИТ2 в зависимости от возраста, индекса массы тела, времени от начала терапии амиодароном.

Определено пороговое значение для возраста: при ≤60 лет прогнозировался высокий риск развития АмИТ2 (AUC 0,715±0,055 с 95% ДИ: 0,607–0,822; р<0,001; чувствительность 69%, специфичность 64,2%). Оптимальным разделяющим значением для ИМТ стал ≤26,6 кг/м² (AUC 0,681±0,058 с 95% ДИ: 0,568–0,793; р=0,003; чувствительность 69%, специфичность 69,5%). Для времени возникновения АмИТ2 от начала терапии Ам определено ≥75,5 недели (AUC 0,643±0,063 с 95% ДИ: 0,519–0,767; р=0,020; чувствительность — 69%, специфичность — 51,6%).

Оценка комплексного влияния факторов на риск развития АмИТ2 произведена с помощью регрессии Кокса. Получена статистически значимая модель пропорциональных рисков (р=0,009), согласно которой возраст ≤60 лет сопровождается ростом риска возникновения АмИТ2 в 2,4 раза (ОШ=2,352, 95% ДИ: 1,053–5,253; р=0,037), ИМТ≤26,6 кг/м² — в 2,3 раза (ОШ=2,301, 95% ДИ: 1,025–5,165; р=0,043). Значения базового риска развития АмИТ2 для разных временных периодов наблюдения представлены в табл. 4.

**Table:** Таблица 4. Значения базового риска развития АмИТ2 для разных временных периодов (максимальный срок – 240 недель)

Временные периоды, недели	Значения базового риска h0(t)
26 недель	0,003
52 недели	0,014
78 недель	0,053
104 недели	0,134
156 недель	0,325
182 недели	0,405
208 недель	0,518
240 недель	0,672

## Обсуждение

Исследование было направлено на изучение распространенности и факторов риска развития АмИТ2 у пациентов с подтвержденным эутиреозом до начала терапии амиодароном, проживающих в районе легкого и умеренного йодного дефицита (медианная концентрация йода в моче в Москве — 67,0 мкг/л, в Московской области — 52,5 мкг/л) [45][46]. В полученной структуре амиодарон-индуцированной дисфункции отмечено преобладание АмИТ2. В представленной выборке за период наблюдения 1–4,6 года частота развития тиреотоксикоза составила 23,4% (29/124) с учетом субклинических форм (5,6% (7/124)), что соответствует высоким значениям диапазона, встречаемого в научных публикациях [30][32][41][47]. АмИТ1 в нашем исследовании развился только у двух пациентов (1,6%): в одном случае — субклинический вариант, через 6 месяцев от начала терапии у пациента с исходно низкореференсным значением ТТГ (0,94 мкМЕ/мл), многоузловым зобом и отсутствием гиперваскуляризации при доплерографии. Длительность приема препарата составила 1 месяц, замена произведена по кардиологическим показаниям. По результатам сцинтиграфии выявлены очаги гиперфиксации пертехнетата (индекс захвата 2,8%), в дальнейшем пациенту выполнена радиойодтерапия. Во втором случае у пациентки с интактной щитовидной железой через 6 месяцев развилась болезнь Грейвса, в течение 1,5 года проводилась тиреостатическая терапия тиамазолом с сохранением ремиссии в течение последующего двухлетнего периода наблюдения, прием амиодарона продолжался по кардиологическим показаниям. Частота амиодарон-индуцированного гипотиреоза составила 19,4% (24/124), сопоставима с предыдущими российскими исследованиями 19,2–19,5% [35][37]. Манифестная форма гипотиреоза, потребовавшая назначение левотироксина, зарегистрирована в 4,8% (6/124) случаев.

Влияние йодного статуса на превалирование АмИТ (йододефицитные регионы) или АмИГ (регионы с адекватным потреблением йода) дискутабельно. Согласно различным исследованиям, отмечается сходная частота АмИТ в йододефицитных 6,9–20,5% [29][30][36] и районах с достаточным обеспечением йода 5,8–30% [26][48][49], что предполагает преобладание других патогенетических механизмов (изменение ауторегуляции щитовидной железы, прямой цитотоксический эффект на тиреоциты, полиморфизм генов, участвующих в биосинтезе и метаболизме тиреоидных гормонов) [19][50].

Отраженная в публикациях частота развития АмИТ в течение первого года приема амиодарона 4,0–5,8% [25][35][40] сопоставима с результатами нашего исследования — 4,8% (6/124). Выявленная высокая частота АмИТ2, наиболее вероятно, обусловлена отбором пациентов на терапию антиаритмиком (определение исходной функциональной и структурной патологии щитовидной железы, строгие критерии включения), проспективным характером исследования, частотой проводимого мониторинга (сокращение интервала оценки тиреоидного профиля с 6 до 3 месяцев).

Несмотря на внушительные выборки в ретроспективных исследованиях иностранных коллег, данные о распространенности АмИТ2 достаточно скудные, в большинстве работ представлены обобщенные результаты, отсутствует разделение по типам АмИТ [30][31][43][47][51]. Зарегистрированная частота манифестного АмИТ2 варьирует от 5,8 до 18,3% [26][29][41]. Возможно, занижение данных истинной распространенности АмИТ2 обусловлено ретроспективным характером исследований. Отсутствие активного регулярного мониторинга функции щитовидной железы приводит к потере части информации — пропуску субклинических и вариантов легкого, реже умеренного течения в связи с нередкой стертостью клинических проявлений, отсутствием классических симптомов тиреотоксикоза (антиадренергическая активность амиодарона и его блокирующее влияние на конверсию Т4 в Т3), доминированием сердечно-сосудистых расстройств (рецидивирование нарушений ритма сердца, учащение ангинозных приступов, появление или усиление признаков хронической сердечной недостаточности) и психоэмоциональной лабильности, не всегда выражена корреляция между уровнями циркулирующих тиреоидных гормонов и клинической тяжестью тиреотоксикоза [40][51][52].

Анализ структуры имеющихся российских ретроспективных исследований случай-контроль показывает включение значительного количества пациентов со структурными изменениями щитовидной железы (50–62,5%), отсутствием данных доплерографии до назначения амиодарона [38][53][54], возможно, с латентными нарушениями функции (исходный уровень ТТГ 1,9±0,1 (0,19-4,5)) [55] и соответствующее превалирование АмИТ1. Распространенность АмИТ2 составила 1,7–9,0% [37][47][55]. Существующие проспективные исследования с частым мониторингом тиреоидного профиля характеризуются небольшой выборкой (до 50 человек), недлительным периодом наблюдения (6–12 месяцев) [53][55], которого недостаточно для представления об истинной распространенности АмИТ2 в связи с нелинейным ее изменением с течением времени, достижением максимума ко 2–3 году и снижением до очень низких показателей после 5-го года приема амиодарона [28][32][41][48]. Медиана развития АмИТ2 в нашем исследовании 92,0 [ 69,0; 116,0] недели сопоставима с результатами Bogazzi F. с соавт. 26,1±24 месяцев [34]; Tomisti L. с соавт. 28,7±16,1, 95% ДИ 27–32 месяцев [32]; среднее время бессобытийной выживаемости 150,2±12,6 недель (95% ДИ 125,5–175,0) недели с результатами Takeuchi D. с соавт. 39 месяцев (диапазон 18–110) [41].

Преобладание в структуре АмИТ 2 типа было продемонстрировано Bogazzi F. с соавт.: в ретроспективной выборке 215 пациентов увеличение среднегодового числа новых случаев АмИТ2 за 27-летний период с 2,4 до 12,5, составляя 89% случаев АмИТ, изменение АмИТ1 с 3,6 в начале периода исследования до 2,5 в последующие годы [34]; Tomisti L. с соавт. в когорте, включавшей 200 пациентов, 79% случаев АмИТ [32]; Takeuchi D. С соавт. при наблюдении 131 пациента все случаи АмИТ были обусловлены деструкцией щитовидной железы [41], и другими авторами [26][28][29].

Неожиданная, непредсказуемая манифестация тиреотоксикоза без предшествующего субклинического нарушения функции щитовидной железы [30][31] имела отражение и в нашей выборке. У 7/29 (24,1%) пациентов развитие тиреотоксикоза произошло между интервалами динамического контроля тиреоидного профиля. Умеренное и тяжелое течение тиреотоксикоза отмечалось у трети пациентов — 31,0% (9/29). Зарегистрированные максимальные значения свободных фракций тиреоидных гормонов сопоставимы с данными Tomisti L. с соавт. 41,7±16,4 и 10,2±5,2 пг/мл [32]; Takeuchi D. С с соавт. 8,02 (2,61–19,2) пг/мл и 4,82 (2,21–7,77) нг/дл [41] для свТ4 и свТ3 соответственно. Клиническую значимость субклинического тиреотоксикоза необходимо учитывать в когорте тяжелых коморбидных пациентов, в связи с увеличением риска нежелательных сердечно-сосудистых событий [56–58].

Молодой возраст как предиктор АмИТ указывается в значительном количестве исследований [25–27][37][59]. Точные пороговые значения были определены лишь в двух: Uchida T. с соавт. — менее 63,5 лет, чувствительность 70,3%, специфичность 69,2% (225 пациентов) [26] и Ahmed S. с соавт. — менее 62 лет (303 пациента) [27]. В нашей выборке также подтверждена зависимость развития АмИТ2 от возраста, разделяющим значением стал менее или равный 60 лет.

Липофильностью и концентрационно-зависимой цитотоксичностью амиодарона объясняется высокий риск развития АмИТ при низком ИМТ. Stan M. с соавт. при разработке индекса прогнозирования риска АмИТ у пациентов с врожденным пороком сердца в качестве предиктора определен ИМТ<21 кг/м² [28]. В нашем исследовании статистическая значимость получена при более высоком значении ИМТ≤26,6 кг/м².

Тяжесть ХСН (II–III ФК NYHA), порока сердца предполагает усиление роли хронической гипоксии в клиническом развитии АмИТ2 в дополнение к непосредственному цитотоксическому действию амиодарона [51]. Однако эти факторы не оказались значимыми в нашей выборке, вероятно, в связи с низкой репрезентативностью данных нарушений в представленной когорте, достаточной компенсацией имеющейся сердечно-сосудистой патологии у большей части пациентов. Наличием хронического воспалительного процесса в миокарде, при котором повышается восприимчивость к окислительному стрессу, вызванному приемом амиодарона [33], можно объяснить большую частоту развития АмИТ2 при миокардите. Не наблюдалось кумулятивное дозозависимое увеличение частоты тиреотоксикоза [60], как и «доза-эффект» у пациентов с ХСН [39]. Патогенетическая взаимосвязь высокой частоты АмИТ с гипотиреозом, развившимся на фоне терапии амиодароном, остается неясной [60], также не прослеживалась в нашей выборке.

АмИТ2 остается важной клинической проблемой, способной вызвать серьезные неблагоприятные сердечно-сосудистые события и быть резистентной к терапии. Предварительное прогнозирование, ранняя диагностика позволят обеспечить превентивные меры по предотвращению клинического ухудшения, затратив дополнительные усилия на контроль ритма и частоты сердечных сокращений [2][61].

## Клиническая значимость результатов

Полученные результаты позволяют определить адекватную тактику ведения пациента.

## Ограничения исследования

Ограничения исследования связаны с недостаточным объемом выборки, необходимостью более длительного периода мониторинга функции щитовидной железы на фоне приема и после отмены амиодарона для повышения релевантности данных, диагностической эффективности полученных прогностических моделей.

## Направления дальнейших исследований

В дальнейшем планируется доработка результатов на большей выборке и валидация.

## Заключение

В проведенном исследовании впервые произведен обширный комплексный анализ факторов, являющихся триггерами развития АмИТ2. Получены новые данные о структуре причин и ключевых конфаундерах. Впервые установлено, что развитию АмИТ2 предрасполагает комбинация клинических предикторов, наиболее значимыми из которых являются возраст, индекс массы тела, а также время от начала терапии амиодароном, детерминирующее реализацию эффекта. Определены сроки развития и распространенность, показана большая частота и преобладание АмИТ2 над другими типами АмИТ.

Получены данные фундаментального характера, расширяющие представления о патогенезе и понимание тематики, обосновывающие возможность использования выявленного сочетания для персонифицированной предикции и оптимизации профилактических и лечебных мероприятий. Предложена новая научная концепция быстрого и доступного прогнозирования риска развития АмИТ2, имеющей огромное значение у пациентов с тяжелой сердечно-сосудистой патологией, позволяющая взвешенно, рационально подойти к выбору антиаритмической терапии и определению адекватного алгоритма мониторинга функции щитовидной железы в каждом конкретном случае.

## Дополнительная информация

Источники финансирования. Работа выполнена по инициативе авторов без привлечения финансирования.

Конфликт интересов. Авторы декларируют отсутствие явных и потенциальных конфликтов интересов, связанных с содержанием настоящей статьи.

Участие авторов. Ермолаева А.С. — сбор и обработка материала, формирование электронной базы данных, статистическая обработка данных, анализ полученных результатов, написание основного текста статьи; Фадеев В.В. — научное руководство проводимого исследования, редактирование и финальное утверждение рукописи. Все авторы одобрили финальную версию статьи перед публикацией, выразили согласие нести ответственность за все аспекты работы, подразумевающую надлежащее изучение и решение вопросов, связанных с точностью или добросовестностью любой части работы.
